# Which actions contribute to the development of an interprofessional learning and working culture in nursing homes? A realist action study

**DOI:** 10.1136/bmjopen-2024-085096

**Published:** 2024-09-20

**Authors:** Frank H O Verbeek, Elvira S Nouwens, Marleen Hermien Lovink, Getty Huisman-de Waal, Cornelia R M G Fluit, Miranda Laurant, Anneke J A H van Vught

**Affiliations:** 1 Organisation of Care and Services, HAN University of Applied Sciences, Nijmegen, The Netherlands; 2 Radboud Institute for Health Sciences, Department of Primary and Community Care, Radboud University, Nijmegen, The Netherlands; 3 IQ Healthcare, Department of Nursing Sciences, Radboud University, Nijmegen, The Netherlands; 4 Research on Learning and Education, Radboud University, Nijmegen, The Netherlands

**Keywords:** Interprofessional Relations, Patient-Centered Care, Quality Improvement

## Abstract

**Abstract:**

**Objective:**

Insights about what actions contributed to the development of an interprofessional learning and working culture were lacking for nursing homes. This study aimed to provide insight into the context and actions that trigger mechanisms for the development of an interprofessional learning and working culture in nursing homes.

**Study design:**

Realist evaluation action research was conducted from 2019 to 2023.

**Setting and participants:**

11 teams in 6 Dutch nursing homes.

**Primary and secondary outcome measures:**

Questionnaires, focus group interviews and observations were used to identify actions, context factors and mechanisms. We used retroductive analysis to discuss how actions were related to the development of the culture. Actions were evaluated in terms of context and manner in which they contribute to the development.

**Results:**

21 actions were identified and clustered into two themes. Theme 1: improving person-centred care. Actions activated the mechanisms of critical reflective behaviour and collective ownership in a context of, among other things, clear roles and tasks, a stable and competent team, the presence of case managers and facilitating organisational factors such as time for reflection. Theme 2: getting to know and understand each other’s expertise. Actions activated respectful relationships, collective ownership of goals and feeling appreciated for your work in a context of, among other things, team members who meet regularly and management supporting interprofessional working.

**Conclusions:**

This research sheds light on how and in what manner-specific actions contribute to the development of an interprofessional learning and working culture in nursing homes. Depending on the context, the actions triggered the following mechanisms: critical reflective behaviour, collective ownership of goals, respectful/caring relationships and feeling appreciated for your work. These mechanisms are the underlying drivers of an interprofessional learning and working culture. This study provides valuable guidance for fostering collaborative and effective interprofessional dynamics in nursing homes.

**STRENGTHS AND LIMITATIONS OF THIS STUDY**

- The design realist action research allowed the teams to select and create actions tailored to their specific context and needs.
- A founder of the realist evaluation approach was consulted to discuss the study’s methodology and proper data analysis.
- Due to the COVID-19 measures, it was not always possible to be physically present in the nursing homes for coaching on the job.
- The questionnaires probably lacked sufficient power to detect significant differences between the beginning and the end of the study.

## Introduction

 In the Netherlands, most older people receive care at home from primary healthcare professionals and informal caregivers. When it is no longer possible to receive this care at home, people with more complex needs are admitted to nursing homes.^1 2^ To meet more complex needs, it is important to work together in an interprofessional way to provide the best quality of person-centred care for nursing home residents.^3^ In person-centred care, the residents and their families become partners with professionals in their own care. The focus is on shared decision-making, emotional well-being and personal goals of the resident, rather than on the illness.

Delivering person-centred care requires intensive collaboration between the resident, their relatives, their informal network and the usually many professionals involved. In the Netherlands, professionals in a nursing home are organised into separate teams, the nursing professionals and allied/medical healthcare professionals. Nursing teams are a mix of nurse aides, nurse assistants, licensed practical nurses, vocationally trained registered nurses and baccalaureate-educated registered nurses at levels 1–6 of the European Qualifications Framework (EQF).^4^ Allied/medical healthcare professionals include physiotherapists, dieticians, speech therapists and general or elderly care physicians. Each professional has their own expertise and competencies in the care of residents. Previous studies have found that the development of an interprofessional learning and working culture enhances the quality of person-centred care of residents.^5^,^8^ An interprofessional learning and working culture is defined as a culture in which different healthcare professionals work intensively and learn together, share an integral vision, set common goals and have responsibilities that cross over into each other’s field.^9^,^12^ However, due to the organisation of daily care or lack of time, we see that most healthcare professionals work within their own field, rely on their own expertise, share little knowledge with colleagues and are not always aware of each other’s expertise in daily practices.^13^ To address these challenges and improve the quality of person-centred care in nursing homes, it is important to facilitate and stimulate interprofessional learning and working.

Recent reviews of interprofessional learning and working cultures in different healthcare settings show that there are many interventions, actions and facilitators to improve interprofessional learning and working. Interprofessional learning and working leads to high standards of care of residents, exemplified by an increase in optimal processes, motivated professionals and involvement of residents and their families in care.^3^ However, there is a gap of knowledge about what works, in what context and in what manner it works in daily nursing home practices. There is, therefore, a need to identify which actions contribute to the development of an interprofessional learning and working culture in nursing homes and to what extent. It is not yet clear what works, in what context and manner it works in daily nursing home practices. This study, therefore, aims to provide insight into the context and actions that trigger mechanisms for the development of an interprofessional learning and working culture in nursing homes.

## Methods and analysis

### Design

**Figure 1:**
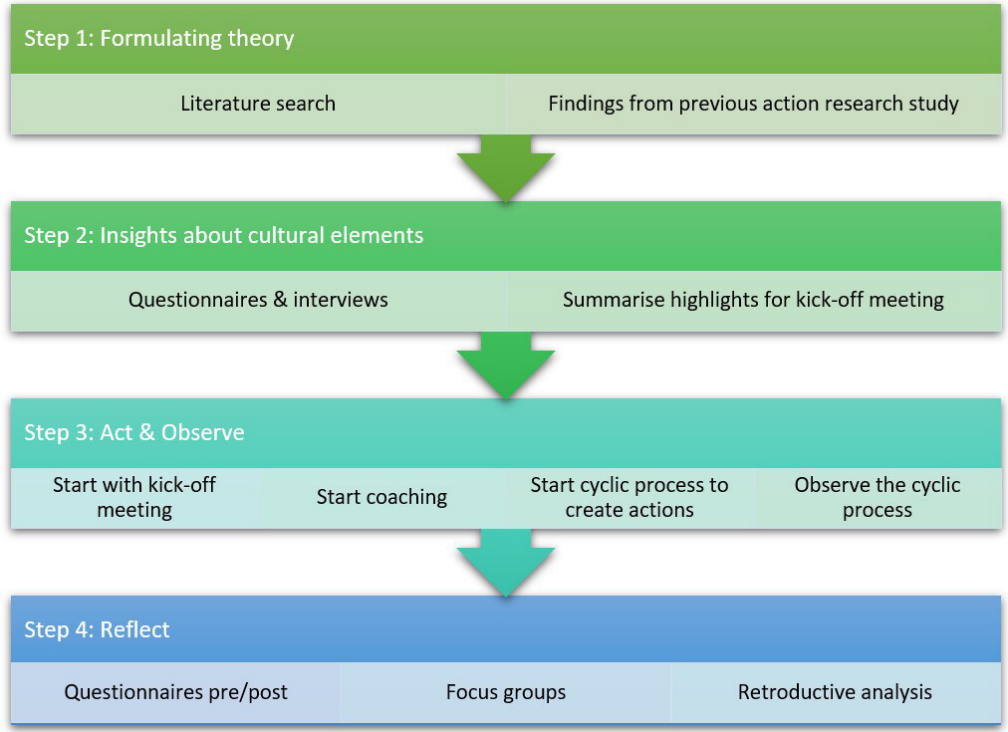
Overview of the four steps in this research.

In order to gain more insight into the context of different nursing homes, what actions and mechanisms are used to develop an interprofessional learning and working culture in nursing homes, the realist evaluation approach was combined with action research.^14^ This realist action research was carried out from September 2019 to July 2023 and consisted of four steps: (1) formulating theory, (2) insights about cultural elements, (3) act and observe and (4) reflect (see figure 1). More details of these steps can be found in the published study protocol.^15^ We used the Rameses II reporting standards for realist evaluation.^16^

### Setting

11 interprofessional healthcare teams participated in this research. The teams worked at six nursing homes: three nursing homes with one participating team, two nursing homes with two teams and one nursing home with four teams. The participating nursing homes, located in the south-east of the Netherlands, specialised in providing healthcare in the area of psychogeriatrics, gerontopsychiatry, rehabilitation, Huntington’s disease, Korsakov syndrome and short stay or observation for older residents.

### Participants

The interprofessional teams consisted of nursing team professionals and allied/medical professionals from levels 2–8 of the EQF.^4^ The number of professionals in the teams varied widely between the units. In the Netherlands, each unit in a nursing home, such as a geriatric rehabilitation unit, has a nursing team. Usually, only allied/medical healthcare professionals work on more than one unit. The allied/medical healthcare professionals were included in the study when they provided care to residents in the participating unit.

### Step 1: formulating theory

#### Initial theory of interprofessional learning and working culture in nursing homes

This study started with the formulation of the initial theory, which could be further developed during the research. This theory was formulated with experts on interprofessional learning and working, the project leader, the researcher and two external coaches. The theory consisted of the description of context-related factors, mechanisms and outcomes.^17^ In each context, mechanisms could be triggered to develop an interprofessional working and learning culture in nursing homes (outcome). In this study, we were interested in the actions that interact with the context and trigger mechanisms to develop the interprofessional working and learning culture. An action is defined as an activity, task or movement that contributes to the main goal, the development of an interprofessional learning and working culture in nursing homes. This initial theory was based on a comprehensive literature search and a previous action research in nursing homes about the development of an evidence-based nursing culture in nursing teams.^13^

##### Summary of the initial theory

To develop an interprofessional learning and working culture, it is important to have insights into the conditions (the context) that are relevant to the operation of the mechanism.^18^ The interprofessional learning and working culture is a culture in which at least two healthcare professionals collaborate and learn together. Innovating, communicating, keeping each other informed, being aware of each other, sharing compliments, sharing successes and collaborating with the residents and their families are needed in this culture. Outcomes could affect at resident, team and organisational level.^14 15 17^ Important conditions are the individual professional factors, team factors, organisation factors, factors of the resident and research/social, political or legal factors. For example, the nursing professionals collaborate with allied and medical healthcare professionals. Each team of professionals (nursing, allied or medical team) has its own culture and mostly works independently of other teams. This could hinder interprofessional collaboration. Furthermore, we identified three mechanisms that enhance the effect of the interprofessional learning and working culture. These are (1) critically reflective work behaviour, (2) collective ownership and (3) respectful/caring relationships. For example, in order to work intensively with each other, it is important to reflect on daily work processes or to respect each other.^19^ More in-depth information is presented in our published research protocol.^15^

### Step 2: insight about cultural elements

First, we created an overview of the presence of the interprofessional cultural elements in each participating team at the start of the research. The insights were discussed in each team in a kick-off meeting and during the team coaching.

#### Measuring the interprofessional cultural elements

To gain an initial overview of the culture, we sent two questionnaires to all healthcare professionals in the interprofessional teams at the starting point of the study. To get more in-depth details, we performed interviews.

##### Questionnaires

The Critically Reflective Work Behaviour Survey^20^ and the Interprofessional Collaboration Measurement Scale^21^ were used. The questionnaires were conducted online with Lime Survey V.3.22.17. The link to the questionnaire was distributed by the contact person in the nursing homes. The questionnaires, including the demographic data of the participants, were descriptively analysed with IBM SPSS Statistics V.27.

##### Interviews

We performed 47 individual interviews with healthcare professionals from the different interprofessional teams to gain an initial overview of the interprofessional learning and working culture at the start of the research. The development of the topic guide was based on relevant outcomes from the earlier action research^13^ and discussions with experts in interprofessional collaboration. Each interview was only summarised by the researcher (FHOV). Highlights of these summaries were recorded to provide an overall picture of the interprofessional learning and working culture in each participating team. These highlights were only used to present and discuss in the kick-off meetings in each participating team (see step 3).

### Step 3: act and observe

Highlights of the questionnaires and interviews were discussed in a kick-off meeting in each participating team. This meeting was the starting point for each team to develop the interprofessional learning and working culture within their own unit. Each team was supported by at least one internal coach, that is, a healthcare professional from within the team. The internal coaches were employees of the nursing team, the allied/medical healthcare team or a combination of both teams. The internal coach or coaches per participating team were selected for their motivation, leadership skills and reflective capacity to coach an interprofessional team in working and learning together in an interprofessional way. The internal coach was coached by two external coaches who were members of the research team and employees of HAN University of Applied Sciences in the Netherlands. The external coaches were experienced lecturers in nursing and allied/medical healthcare and had expertise in culture change, the nursing home setting and interprofessional learning and working. These coaches worked with the practice development approach (PD) to support and encourage professionals to work together and to involve residents and family members. Residents and family members, together with professionals, selected and performed the actions to encourage interprofessional work contributing to person-centred care. The PD approach focused on creating working and learning cultures and on developing person-centred cultures. The PD approach consisted of nine principles, for example, focusing on microlevel and working with short cyclic innovation processes in the workplace.^22 23^ Highlights of the questionnaires, interviews and the discussion in the kick-off meeting were subsequently used to identify topics for the development of an interprofessional learning and working culture. The internal coaches followed a cyclic process at the team level, which means that they started by identifying topics for developmental action.^23^ The cyclic process consisted of six processes (called PDDOEN in Dutch): (1) creating an overview of a subject, the need, the why and who is involved, (2) setting a goal to improve interprofessional learning and working, (3) selecting or creating the needed actions, (4) sharing and collecting new insights, (5) evaluating and (6) selecting/creating actions or selecting a new topic.^23^ During this cyclic process, internal and external coaches and the researcher (FHOV) observed and identified actions occurring in the participating teams. Observations were recorded in a logbook. During the action and observation period, we had several meetings with the internal coaches, the external coaches and the researcher (FHOV) to share ideas, inspiration and discuss the process.

### Step 4: reflect

This step was about getting insights into what works (actions), and in what context and what manner (mechanism). We used the outcomes of the two questionnaires that were repeated at the end of the study (described in step 2), the logbooks (described in step 2) and the focus group sessions per participating team at the end of the study.

All the questions in the two questionnaires were categorical and were tested with the χ^2^ test to analyse whether the statements (agree–disagree) changed between the start and the end of the study. The level of significance was established at p=0.05.

The aim of the focus group sessions was to create insight into the actions, the influence of the context and how these actions worked. A total of 4–10 professionals participated in each focus group; nursing team members and allied/medical healthcare professionals were mixed together. The focus group sessions were audio-taped and transcribed. The transcriptions were deductively analysed using the subjects from the initial theory with Atlas TI by two researchers (FHOV and ESN). Findings from the analysis were discussed between these two researchers and with the project leader (AJAHvV). We created a summary text for each participating team for members to check.

For the overall analysis, we used iterative steps based on retroductive analysis; identification of the actions; exploration of what works, in what context and how actions influenced mechanisms in a specific context that contribute to the outcome; and lastly formulation of the relationship between context, mechanism and outcome.^24^

#### Identification of the actions

Actions were identified by screening the logbooks and discussing the identified actions in the focus group sessions. After identification, the actions were clustered into themes of actions through the discussions with the researchers (FHOV, ESN), external coaches (ESN, AW) and the project leader (AJAHvV).

#### Exploration

To explore how these actions were related to the development of the culture, FHOV and ESN deductively analysed the focus group transcripts independently for the performed actions, the context factors, which mechanisms were triggered and what outcomes were seen. After that, FHOV, ESN and AJAHvV explored and reported which patterns in terms of context, mechanism and outcome on the interprofessional learning and working culture were seen in the themes of actions.

#### Formulation

Lastly, we discussed the patterns of what works, in what context and how with a group of experts on interprofessional collaboration in healthcare and with the internal coaches who participated in the study. These discussions were held by means of an online meeting and bilateral conversations. Such broad discussion enabled us to formulate and refine the relationship between the context, mechanism and outcome in the themes of actions.

## Results

### Demographic data

The demographic data from the questionnaires and focus groups are presented in table 1. In total, 128 professionals completed the two questionnaires at the start of the research and 66 at the end of the research. Of these, 58.6% (pre) and 65.2% (post) were members of a nursing team and most were female (93% pre, 87% post) and with a mean age of 40–42 years. In addition, 34 healthcare professionals were interviewed in 7 focus group sessions with a mean duration of 70 min. 19 professionals were members of a nursing team and 32 professionals were female, mean age was 42 years.

**Table 1:** Demographic data of participants, questionnaires and focus groups

Demographic data—n (%)					
Questionnaires pre (n=128)		Questionnaires post (n=66)		Focus groups (n=34)	
Gender		Gender		Gender	
Female	119 (93%)	Female	58 (87.9%)	Female	32 (94.1%)
Male	9 (7%)	Male	7 (10.6%)	Male	2 (5.9%)
Other	.	Other	1 (1.5%)	Other	.
Age, years		Age, years		Age, years	
Range	18–63	Range	22–61	Range	24–65
Mean (SD)	42 (12.568)	Mean (SD)	40 (11.439)	Mean (SD)	42 (12.299)
Work experience (years)		Work experience (years)		Work experience (years)	
<1	8 (6.3%)	<1	12 (18.2%)	Range	0–34
1–3	17 (13.3%)	1–3	8 (12.1%)	Mean (SD)	10 (10.461)
4–6	15 (11.7%)	4–6	5 (7.6%)		
7–9	7 (5.5%)	7–9	7 (10.6%)		
10–12	13 (10.2%)	10–12	6 (9.1%)		
13–15	6 (4.7%)	13–15	28 (42.4%)		
≥16	62 (48.4%)	≥16			
Educational level*		Educational level*		Educational level*	
EQF 2	9 (7.1%)	EQF 2	2 (3%)	EQF 2	1 (2.9%)
EQF 3	39 (30.5%)	EQF 3	14 (21.2%)	EQF 3	5 (14.7%)
EQF 4–5	17 (13.3%)	EQF 4	15 (22.7%)	EQF 4	3 (8.8%)
EQF 6	39 (30.5%)	EQF 6	25 (37.9%)	EQF 6	19 (55.9%)
EQF 7–8	17 (13.3%)	EQF 7–8	7 (10.6%)	EQF 7–8	6 (17.6%)
Other	4 (4.7%)	Other	3 (4.5%)	Other	.
Missing	1 (.8%)	Missing	.	Missing	.
Organisation		Organisation		Organisation	
Organisation 1	15 (11.7%)	Organisation 1	14 (21.2%)	Organisation 1	4 (11.8%)
Organisation 2	42 (32.8%)	Organisation 2	14 (21.2%)	Organisation 2	8 (23.5%)
Organisation 3	19 (14.8%)	Organisation 3	10 (15.2%)	Organisation 3	8 (23.5%)
Organisation 4	13 (10.2%)	Organisation 4	8 (12.1%)	Organisation 4	2 (5.9%)
Organisation 5	12 (9.4%)	Organisation 5	15 (22.7%)	Organisation 5	6 (17.6%)
Organisation 6	27 (21.1%)	Organisation 6	5 (7.6%)	Organisation 6	6 (17.6%)
Belongs to:		Belongs to:		Belongs to	
Nursing staff	75 (58.6%)	Nursing staff	43 (65.2%)	Nursing staff	19 (55.9%)
Allied/medical staff	47 (36.7%)	Allied/medical staff	23 (34.8%)	Allied/medical staff	12 (35.3%)
Manager/researcher	.	Manager/researcher	.	Manager/researcher	3 (8.8%)
Missing	6 (4.7%)	Missing	.	Missing	.

* EQF levels: EQF 2 basic vocational education and training—EQF 3 advanced vocational education and training—EQF 4 middle management or specialist vocational education and training—EQF 5 associate degree—EQF 6 bachelor’s degree—EQF 7 master’s degree—EQF 8 medical specialist (doctorate).^37^

EQFEuropean Qualifications Framework

14 internal coaches and 2 external coaches participated in this study. Two internal coaches in organisation 1 (belonging to the allied/medical staff), one internal coach in organisation 2 (belonging to the nursing staff), four internal coaches in organisation 3 (two belonging to the nursing staff and two belonging to the allied/medical staff), two in organisation 4 (one belonging to the nursing staff and one to the allied/medical staff), three in organisation 5 (two to the nursing staff and one to the allied/medical staff) and two internal coaches were participating in organisation 6 (belonging to the nursing staff).

### Brief reflection on initial theory

The initial theory is presented as hypotheses under the heading’s context, mechanisms and outcomes. Context factors in interprofessional learning and working as mentioned in the initial theory emerged to a greater or lesser extent in this study, except in relation to COVID-19 measures. In the Netherlands, all nursing homes for the elderly were closed to visitors, family and certain allied/medical healthcare professionals who were not involved in the basic care for the elderly. This measure played a major role in interprofessional learning and working because there were fewer physical meetings with the interprofessional teams, and less attention given to interprofessional working and more to the COVID-19 pandemic.^25^ The pandemic made it difficult to meet up, but it also improved collaboration and learning around care-related themes such as loneliness or stimulus processing. With respect to the mechanisms, three mechanisms reported in the initial theory were confirmed in this study. In addition, we identified a fourth mechanism that influenced the interprofessional learning and working culture: ‘feeling appreciated for your work’. Described outcomes, such as intended and unintended consequences of the actions, corresponded to the initial theory.

In this study, we were particularly interested in the actions that trigger mechanisms in a specific context and result in the intended outcomes. This study integrated actions in the initial theory and configured the context-mechanism-outcome around the actions.

### Themes of actions including context-mechanism-outcome configurations

We identified 21 actions that were clustered into 2 themes of actions: (1) improving person-centred care and (2) getting to know and understand each other’s expertise. For each theme of actions, we presented the actions, the context, the mechanisms they triggered in the context and the outcomes in developing an interprofessional learning and working culture.

### Theme 1: improving person-centred care

We distinguished several types of actions to improve person-centred care. Actions focusing on (1) working on care-related themes to solve daily questions in healthcare in a bottom-up way, (2) being aware of the resident’s needs, for example, through a multidisciplinary intake with the resident and all the healthcare professionals and (3) involving family, by using communication tools or motivating residents to use an activity box with their relatives (table 2).

**Table 2:** Actions improving person-centred care

Actions	Outcomes
Working with care-related themes	
Making ideas transparent about what subjects motivate people and what we can develop through using post-it notes on flip charts	Professionals collaborate and learn togetherProfessionals innovateIncreased development of knowledge and skillsCollaboration with residents and their families
Using evidence-based practice	Professionals ask themselves and others critical questionsCelebration of successIncreased development of knowledge and skillsImprovement of person-centred care
Personally approaching individuals for participation in working groups	Increased job satisfaction
Project on stimulus processing	Improvement of person-centred careImprovement of the collaboration with residents and their familiesProfessionals collaborate and learn togetherKeep each other informedCommunicate with an open attitude
Quality improvement project to deal with sense of life	Professionals collaborate and learn togetherCollaboration with residents and their familiesImprovement of person-centred careKeep each other informedMore innovationShare compliments and successesCelebration of success
In-company training on healthcare-related topics	Improvement of person-centred careIncreased development of knowledge and skills
Quality improvement project on coping with delirium	Keep each other informedImprovement of person-centred careProfessionals collaborate and learn together
Qualitative research to measure loneliness among residents	Increased development of knowledge and skillsMore innovationImprovement of person-centred careIncreased job satisfactionProfessionals ask themselves and others critical questionsProfessionals are aware of each other
Being aware of the resident	
Setting up a multidisciplinary intake	Improvement of person-centred careIncreased development of knowledge and skills
Setting up a ‘walking-list’ showing name, reason for admission, advice	Keep each other informedImprovement of person-centred care
Setting up a mini-multidisciplinary consultation	Keep each other informedProfessionals collaborate and learn togetherProfessionals are aware of each other
Working in the triangle consisting of case manager, welfare coordinator and team coach	Improvement of person-centred careProfessionals collaborate and learn togetherKeep each other informedCollaboration with residents and their families
Involving family	
Informing family using online communication systems	Improvement of person-centred careProfessionals collaborate and learn together
Motivating family to use the newly available ‘well-being activity box’ with their relatives	Improvement of person-centred careCollaboration with residents and their family

#### Context

**Figure 2:**
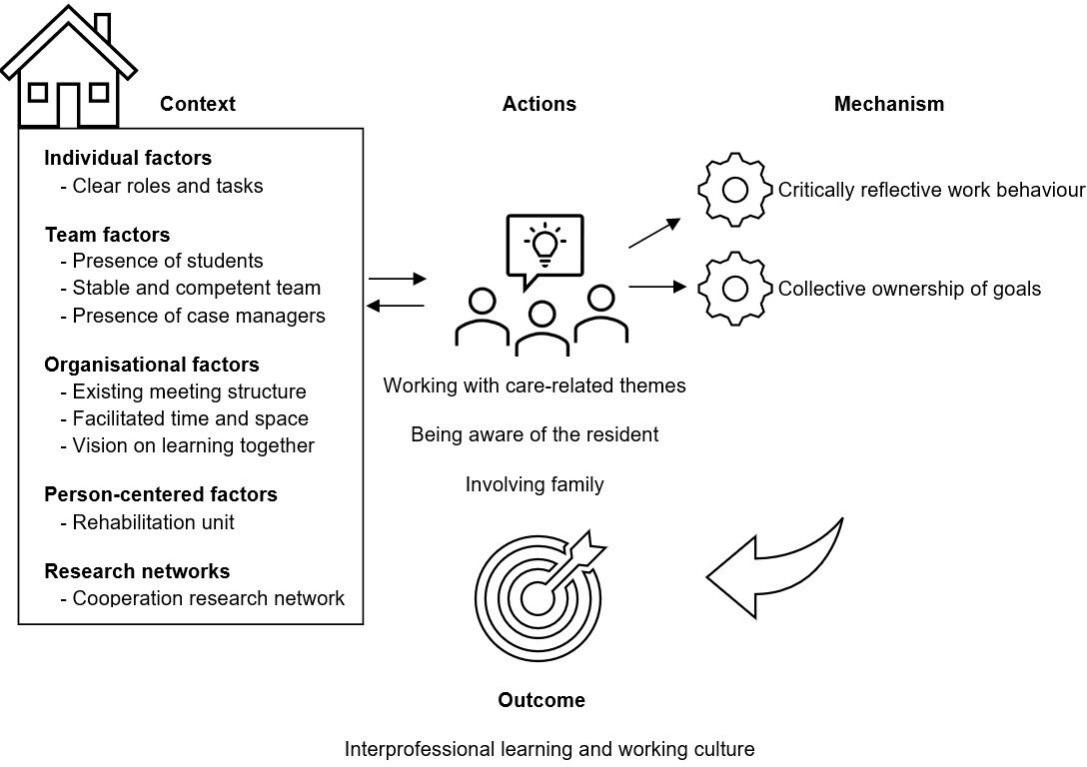
Improving person-centred care.

In this theme of actions, we saw the following context factors (figure 2) that were also presented in our initial theory: (1) team factors, (2) organisational factors, (3) person-centred factors and (4) research network.

Individual professional factors

- Clear professional roles and tasks helped staff to know what to expect from each other.

Team factors

- The presence of students in the units. Students asked critical questions about daily care and working methods. This was helpful to establish a solid cooperation between all healthcare professionals.
- A stable, competent, permanent healthcare team contributed to knowing each other’s competences.
- *‘If you don't have a stable team … yes, you will go under’. (organisation 5*)
- The presence of case managers in a team with a nursing background resulted in a context where it was possible to innovate and be involved together.
  ‘Because we (case managers) have a nursing background and so we also want to be involved in the team in that way. How can we achieve this? By simply joining the medical visits!’ (organisation 6)

Organisational factors

- The presence of an existing meeting structure stimulated meeting each other on a regular basis. We saw that this meeting structure was more prevalent in a care innovation unit or in a geriatric rehabilitation unit compared with other units.
- *‘There is a weekly meeting with the allied/medical team and nursing team together and then a few clients are discussed. I think this meeting allows us to brainstorm and consult with each other in a multidisciplinary way’ (organisation 1*)
- The facilitation in space and time to be an internal coach or to learn and work together as an interprofessional team on projects, for example, working on care-related themes or on (small) evidence-based practice projects. The internal coach coached other healthcare professionals or made a project plan to initiate a project.
  ‘We think this (doing research) is important, so you must be facilitated… If you don’t facilitate, how seriously do you take someone?’ (organisation 1)
- In a context where organisations had a clear vision of learning together, performing research and innovating, interprofessional learning was more embedded in daily practices.

Patient-related factors

- Working on a rehabilitation unit with short cyclic processes, the nursing home residents were more likely to be involved as partners in care to achieve the rehabilitation goals.

Research network factors

- Cooperating with a research network or an academic nursing home network supported the focus on learning together.

#### Mechanism

These actions in these contexts triggered the mechanisms:

Critical reflective behaviour, through conversations about the best person-centred care. Healthcare professionals were learning from mistakes in daily practice, were making suggestions for a different way of working and were experimenting together. The questionnaire on critical reflective work behaviour showed that only 2 of the 47 statements significantly differed between the start and end of the study (online supplemental appendix 1). The first relates to comparing their own performance with the performance of colleagues, which was more frequent at the end of the study (72.4% pre, 89.4% post, p=0.007). Furthermore, there was a small negative outcome for the statement ‘if I make a mistake, I find it hard to forgive myself’. This statement was more frequent at the end of the study (42.7% pre, 62.1% post, p=0.012).

Collective ownership of goals, in which a shared vision emerges, and joint responsibility is felt for reaching and achieving shared goals. For example, by engaging in joint discussion, a care plan is immediately drawn up, including a possible discharge date at rehabilitation. Other examples are actions aimed at working together on an electronic patient record because professionals and residents can read, consult and tag each other’s notes. This made them feel involved and jointly responsible.

#### Outcome

The actions on person-centred care in the above-described context resulted in an improved interprofessional working and learning culture. It appears that there was more collaboration between professionals and with residents and their families. Professionals were innovating, doing research, sharing information and sharing compliments and successes. They were aware of each other’s expertise, were more motivated to improve daily healthcare and there was more focus on the resident’s well-being. The questionnaire, the Interprofessional Collaboration Measurement Scale, showed that 2 of the 13 statements were significant when comparing the pre and post data, indicating improved interprofessional collaboration (online supplemental appendices 2 and 3). Sharing information about residents between the nursing team and allied/medical professionals was more frequent at the end of the research (81%) compared with the start of the research (53.3%), p=0.031. The item of discussing the resident’s care was more frequent (64.4% pre, 95.2% post) at the end of the study period, p=0.000.

### Theme 2: getting to know and understand each other’s expertise

Actions that contribute to knowing and understanding each other’s expertise were seen in the actions (1) getting to know each other and (2) coaching (table 3).

**Table 3:** Actions getting to know and understand each other’s expertise

Actions	Outcomes
Getting to know each other	
Using post-it notes to understand what people think the tasks and responsibilities of each profession are	Professionals collaborate and learn together
Understanding each other’s tasks and responsibilities through vlogs and a discussion meeting	Professionals are aware of each otherKeep each other informed
Getting to know each other in informal ways over coffee or through informal gatherings	Professionals are aware of each otherProfessionals ask themselves and others critical questionsInnovateProfessionals collaborate and learn togetherCommunicate with an open attitude
Leaving office doors open	Professionals collaborate and learn together
Coaching	
Organising daily evaluations	Professionals collaborate and learn togetherProfessionals are aware of each otherCommunicate with an open attitudeImprovement of person-centred careKeep each other informedInnovateShare compliments and success
Organising peer-group coaching/reflection meetings by the external coach	Communicate with an open attitudeShare compliments and successProfessionals are aware of each otherProfessionals ask themselves and others critical questions
The organisation enables allied and medical healthcare professionals to work in one unit rather than across different units	Professionals collaborate and learn togetherKeep each other informedImprovement of person-centred care

#### Context

**Figure 3:**
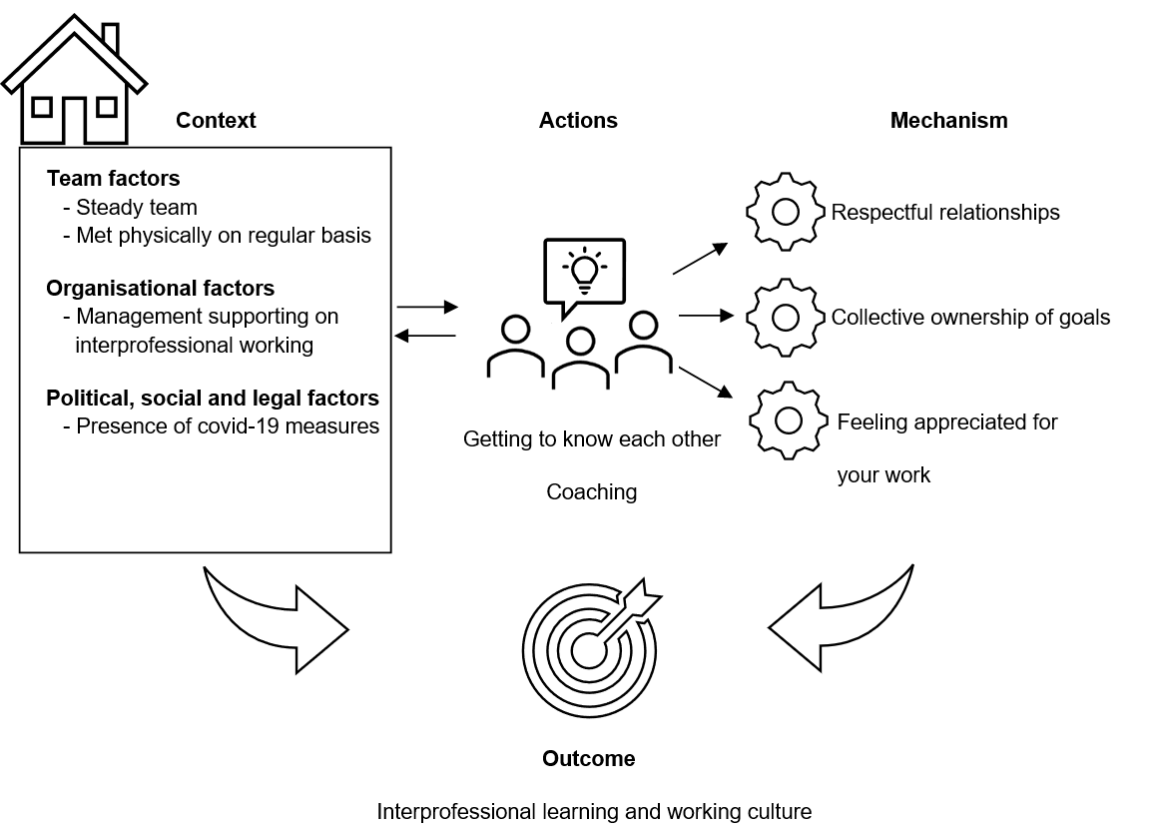
Getting to know and understand each other’s expertise.

In this theme of actions, we identified the following context factors (figure 3) that were also presented in our initial theory: (1) team factors, (2) organisational factors and (3) social, political and legal factors. We also found a new context factor, the presence of COVID-19 measures. We saw this context factor as a ‘social, political and legal factor’ because the Dutch government implemented the COVID-19 measures for the Netherlands during the pandemic.

Team factors

- There were reasonably stable teams in which healthcare professionals had considerably large contracts. This meant there were not too many changes in the team structure and it was easier to get to know each other.

Organisational factors

- Interprofessional teams that met physically on a regular basis helped to create a safe environment, as a result of more informal contact.
- *‘And I also think that when you see each other at work … That you know what the other person is doing. Sometimes it’s a bit vague what someone is doing, but if you see it literally then you also know what you have to do with that person…more clarity.’ (organisation 3*)
- When management provided support for interprofessional working, such as setting goals for interprofessional working, making time for interprofessional working, discussing interprofessional working and improving interprofessional working, the team felt there was a real opportunity to work together.

Social, political and legal factors

- In some teams, COVID-19 measures meant that professionals (also allied and medical professionals) were less physically present in the units, which made it difficult to meet regularly. On the other hand, it also resulted in teams being more focused together on patient loneliness or stimulus processing.

#### Mechanism

These actions in these contexts triggered the following mechanisms:

Respectful relationships, in which professionals get to know each other better and therefore trust each other more, allowing for greater mutual understanding.

I think that with the peer-group coaching, there was also a bit of, yes… reflection… Okay, so how can this be experienced by another person? (organisation 2)

Collective ownership of goals, in which joint responsibility is triggered among team members for person-centred care.

Feeling appreciated, explained as team members feeling that they are really taken seriously and that their opinions and suggestions are heard. This was expressed, for example, by deliberately asking for everyone’s ideas about the case and using those ideas.

#### Outcome

These actions that focus on knowing and understanding each other‘s expertise helped in developing a learning and working culture. This resulted in team members being more aware of each other’s perspectives and expertise, keeping each other informed and learning together. Questioning each other in an approachable way was easier, as was discussing things together. The actions related to informal meetings resulted in easier ways to support each other, give feedback or make suggestions for improvement.

Approaching each other is easier, because you're there. So, you don't have to go searching. (organisation 3)

We just need each other, and you have to look for each other. You’re looking for the best and most efficient way to help such a resident as best as possible. (organisation 3)

Furthermore, the peer-group coaching and daily evaluations resulted in sharing compliments and successes more often, more communication with an open attitude, learning together and increased job satisfaction.

We walk in on each other, we use each other’s knowledge, we help each other, also the practitioners, and they help us in the care task as we help them. I feel that very much. There’s also a lot of room for consultation over coffee with each other, but also by sparring and consulting with each other on how we can improve our quality of care … (organisation 5)

## Discussion

This research provided insight into the context and actions that trigger mechanisms for the development of an interprofessional learning and working culture in nursing homes. 21 actions were identified. These are clustered into two themes of actions. The first theme of actions was aimed at improving person-centred care. Actions activated the mechanisms of critical reflective behaviour and collective ownership in a context of, among other things, clear roles and tasks, a stable and competent team, the presence of case managers and facilitating organisational factors such as time for reflection. The second theme of action focused on getting to know and understand each other’s expertise. In this theme of actions, the mechanisms of respectful relationships, collective ownership of goals and feeling appreciated for your work were activated in a context of, among other things, team members who meet regularly and with management supporting interprofessional working.

The development of an interprofessional learning culture has been studied extensively, mainly in hospital settings.^26^ A recent study about interprofessional collaboration in hospitals found that building on care relationships and building on constructive feedback were important underlying mechanisms.^19^ The same findings were reported in a recent scoping review about facilitators in the development of an interprofessional learning culture in nursing homes, such as having a safe, respectful and transparent environment or having a frontline manager who facilitates and supports change.^27^ Unfortunately, information on the operationalisation of such facilitators was limited. The present study identified more specific actions, relevant contextual factors and how these actions contribute to an interprofessional learning and working culture in nursing homes. For example, an interprofessional learning and working culture could be developed through critically reflective behaviour. To achieve this, targeted actions can be implemented, such as developing a safe environment in which professionals feel comfortable giving feedback. Actions to achieve this safe environment are ensuring that professionals meet physically on regular basis, organising daily evaluations to give each other feedback and having peer-to-peer reflection meetings with (external) coaches.

Team and organisational context factors played an important role in developing and selecting actions. For example, actions such as getting to know each other better and aiming at a safe environment are more effective in a context in which careful relationships and a stable team can be built. In addition, this research shows that having a stable, competent and permanent team and facilitating collaboration in time and space act as factors for the development of an interprofessional learning and working culture. This is also a challenge because of the shortage of professionals and time in nursing homes in the Netherlands.^28^ Conversely, the presence of an interprofessional learning and working culture works positively on job satisfaction because effective teamwork and shared decision-making are known to be associated with job satisfaction. This is important because job satisfaction contributes to engaging and retaining professionals in a team so that a stable team can be secured.^29^ A clear vision of the organisation and management on learning and interprofessional working is also seen as an important factor to create opportunities for individual professionals and the teams to work with a shared vision in daily practice.^30^ In addition, units such as a learning unit or a geriatric rehabilitation unit with short stays for residents are a good basis for interprofessional learning and working. A lot of students are participating as interns in such units for several months and work on various professional and interprofessional assignments.^31^ This could promote interprofessional learning and working together. The short cycle stays in a geriatric rehabilitation unit, where residents work on rehabilitation goals for an average of 1 month,^32^ also means that professionals must work more intensively together with collective ownership of goals. Rehabilitation is a team approach, involving numerous professionals and the resident.^33^ This setting would seem more conducive to interprofessional working than a psychogeriatric unit in a nursing home. To promote interprofessional working in a somatic or psychogeriatric unit, it is important to invest in space and time to get to know each other and to meet each other on a regular basis.

This study has given us a better understanding of the mechanisms involved in developing an interprofessional learning and working culture. It turned out that four instead of three mechanisms could be triggered by actions for the development of interprofessional learning and working culture. Actions to get to know each other better triggered respectful relationships in teams and actions to trust and joint responsibility triggered more collective ownership of goals. These are important mechanisms because commitment to goals or respectful relationships creates involvement of all the professionals, the residents and their families and could improve person-centred care and a vision about the best person-centred care in the nursing homes.^19 34^ In addition to our presented initial theory, it turned out that feeling appreciated for your work is also important. For example, healthcare professionals are more satisfied when their efforts in daily practices are seen or recognised by team members or the front-line manager.^35^ This increases their motivation to improve the quality of care. In interprofessional collaboration, it is, therefore, important to recognise each professional and their expertise.^26^ The recognition of a professional’s expertise, ideas or knowledge forms an important part of working in a team.^10^

### Strengths and limitations

The strength of this study was its realist action design because it was appropriate for the actions to be selected and created by the teams. The internal coaches coached in daily practice by selecting and creating these actions with the teams. It was, therefore, possible to tailor the actions to the specific contexts and needs of the different participating teams. In the present study, an expert on the realist evaluation approach was consulted to discuss the findings and analysis methods to improve this process and provide the best insights to make implications for the daily nursing home practice.

Some limitations were seen in this study. This study focused on the actions to develop an interprofessional learning and working culture. These actions also improved person-centred care in some way. However, the effect of person-centred care was not measured in this study. Further research should be focused to find how the actions affect the person-centred care experienced by the residents in the nursing homes. Due to the COVID-19 measures, it was not always possible to be physically present in the nursing homes for coaching on the job, to discuss and reflect on the actions and to observe the actions. A physical distance was noticed between the internal coaches and professionals in the units and the external coaches. Despite this, the healthcare professionals continued selecting and creating actions to improve the interprofessional learning culture. It seems that the COVID-19 measures provided more insight into how important it is to communicate, work together, be physically present and achieve the highest quality of person-centred care.^36^ For example, some care-related themes were selected because of the measures, such as loneliness or well-being of the residents. Due to the limited response to the questionnaires, the power of the questionnaires was probably too low to find significant differences between the start and the end of the study. Professionals may have lacked the time to complete the questionnaires due to the COVID-19 measures. For future research, a shorter questionnaire is recommended and perhaps one instead of two questionnaires to increase the response rate.

Furthermore, the influence of coaches’ (personal) characteristics on the development of interprofessional learning culture was not investigated in this study. It is recommended for further research to investigate the (personal) characteristics of the coaches on interprofessional learning and working so that they can be considered when selecting and training coaches to develop an interprofessional learning and working culture.

## Conclusion

This realist action research sheds light on how and in what manner specific actions focused on improving person-centred care and getting to know and understand each other’s expertise contribute to fostering an interprofessional learning and working culture in nursing homes. Depending on the context, the actions triggered four mechanisms: critically reflective behaviour, collective ownership of goals, respectful/caring relationships and feeling appreciated for your work. These mechanisms are the underlying drivers of interprofessional learning and working culture in nursing homes. The findings highlight the significance of prioritising person-centred care and cultivating mutual understanding of diverse expertise. They also highlight the critical influence of contextual factors in cultivating and sustaining such a culture in these healthcare settings. These insights provide valuable guidance for fostering collaborative and effective interprofessional dynamics within nursing homes. It is recommended that interprofessional teams actively invest in promoting an interprofessional learning and working culture by selecting actions appropriate to their context. Further research should focus on the effectiveness and feasibility of the identified actions within specific and different contexts in nursing homes.

## supplementary material

10.1136/bmjopen-2024-085096 online supplemental file 1

## Data Availability

Data are available on reasonable request.
