# Supplementary material for: Which actions contribute to the development of an interprofessional learning and working culture in nursing homes? A realist action study
Source: BMJ Open. 2024 Sep 20;14(9):e085096. doi: 10.1136/bmjopen-2024-085096 (PMC11418486; doi:10.1136/bmjopen-2024-085096)
Supplement: online supplemental file 1 [file bmjopen-14-9-s001.pdf]

Appendix 1 – Survey: Critically reflective work behaviour survey

Statement 1: I reflect on the way of working				
	pre	*post*	*value (df)*	*p-value**
agree	115 (93.5%)	63 (95.%)		.750
disagree	8 (6.5%)	3 (4.5%)		
Statement 2: I think about communication with colleagues				
	pre	*post*	*value (df)*	*p-value**
agree	119 (97.5%)	2 (3.0%)		1.000
disagree	3 (2.5%)	64 (97%)		
Statement 3: I find it hard to pinpoint what I have learned last year				
	pre	*post*	*value (df)*	*p-value*
agree	22 (18%)	10 (15.2%)	.252(1)	.616
disagree	100 (82%)	56 (84.8%)		
Statement 4: I ponder on what I find important in my work				
	pre	*post*	*value (df)*	*p-value**
agree	118 (95.9%)	63 (95.5%)		1.000
disagree	5 (4.1%)	3 (4.5%)		
Statement 5: I compare my organisation with similar organisations				
	pre	*post*	*value (df)*	*p-value*
agree	80 (65.6%)	49 (74.2%)	1.495(1)	.222
disagree	42 (34.4%)	17 (25.8%)		
Statement 6: I compare my performance with how I performed a year ago				
	pre	*post*	*value (df)*	*p-value*
agree	101 (82.1%)	53 (80.3%)	.093(1)	.760
disagree	22 (17.9%)	13 (19.7%)		
Statement 7: I reflect on what I have not done well in the past year				
	pre	*post*	*value (df)*	*p-value*
agree	105 (85.4%)	60 (90.9%)	1.191(1)	.275
disagree	18 (14.6%)	6 (9.1%)		
Statement 8: I compare my performance with my colleagues at work				
	pre	*post*	*value (df)*	*p-value*
agree	89 (72.4%)	59 (89.4%)	7.339(1)	.007
disagree	34 (27.6%)	7 (10.6%)		
Statement 9: I discuss with colleagues how I have developed				
	pre	*post*	*value (df)*	*p-value*
agree	83 (68.6%)	46 (69.7%)	.024(1)	.876
disagree	38 (31.4%)	20 (30.3%)		
Statement 10: I discuss with colleagues why you can't always do this work according to the rules				
	pre	*post*	*value (df)*	*p-value*
agree	99 (81.8%)	56 (84.8%)	.276(1)	.599
disagree	22 (18.2%)	10 (15.2%)		
Statement 11: If I think I have not done my work well, I discuss this with colleagues				
	pre	*post*	*value (df)*	*p-value**
agree	110 (90.9%)	63 (95.5%)		.385
disagree	11 (9.1%)	3 (4.5%)		
Statement 12: If I think I have done my work badly, I discuss this with my supervisor.				
	pre	*post*	*value (df)*	*p-value*
agree	83 (68.6%)	46 (69.7%)	.024(1)	.876
disagree	38 (31.4%)	20 (30.3%)		

Statement 13: I ask my supervisor for feedback

	pre	*post*	*value (df)*	*p-value*
agree	73 (60.3%)	35 (53%)	.933(1)	.334
disagree	48 (39.7%)	31 (47%)		

Statement 14: I ask my colleagues for feedback

	pre	*post*	*value (df)*	*p-value*
agree	106 (87.6%)	57 (86.4%)	.059(1)	.809
disagree	15 (12.4%)	9 (13.6%)		

Statement 15: I ask my customers (internal and external) what they think of my services or products

	pre	*post*	*value (df)*	*p-value*
agree	91 (75.2%)	53 (80.3%)	.626(1)	.429
disagree	30 (24.8%)	13 (19.7%)		

Statement 16: I discuss with my colleagues what I find important in my work

	pre	*post*	*value (df)*	*p-value**
agree	115 (95%)	60 (90.9%)		.350
disagree	6 (5%)	6 (9.1%)		

Statement 17: I invite colleagues to assess my work critically

	pre	*post*	*value (df)*	*p-value*
agree	89 (73.6%)	49 (74.2%)	.010(1)	.918
disagree	32 (26.4%)	17 (25.8%)		

Statement 18: I discuss with my colleagues our criteria for performing well

	pre	*post*	*value (df)*	*p-value*
agree	97 (80.2%)	49 (74.2%)	.875(1)	.350
disagree	24 (19.8%)	17 (25.8%)		

Statement 19: I come up with ideas how things could be organised differently here

	pre	*post*	*value (df)*	*p-value*
agree	107 (90.7%)	60 (90.9%)	.003(1)	.959
disagree	11 (9.3%)	6 (9.1%)		

Statement 20: I make suggestions to my supervisor about a different working method

	pre	*post*	*value (df)*	*p-value*
agree	77 (65.3%)	44 (66.7%)	.038(1)	.846
disagree	41 (34.7%)	22 (33.3%)		

Statement 21: I give my opinion about developments at work

	pre	*post*	*value (df)*	*p-value**
agree	112 (94.9%)	64 (97%)		.713
disagree	6 (5.1%)	2 (3%)		

Statement 22: I call this organisation's policy into question

	pre	*post*	*value (df)*	*p-value*
agree	77 (65.3%)	51 (77.3%)	2.888(1)	.089
disagree	41 (34.7%)	15 (22.7%)		

Statement 23: I put critical questions to my supervisor about the working of this organisation

	pre	*post*	*value (df)*	*p-value*
agree	66 (55.9%)	39 (59.1%)	.172(1)	.678
disagree	52 (44.1%)	27 (40.9%)		

Statement 24: I make suggestions to my colleagues about a different working method

	pre	*post*	*value (df)*	*p-value*
agree	93 (78.8%)	57 (86.4%)	1.602(1)	.206
disagree	25 (21.2%)	9 (13.6%)		

Statement 25: If everyone agrees I will remain critical

	pre	post	value (df)	p-value
agree	99 (83.9%)	53 (80.3%)	.381(1)	.537
disagree	19 (16.1%)	13 (19.7%)		
Statement 26: When I do not agree with the way a colleague works, I just keep it quiet				
	pre	post	value (df)	p-value
agree	24 (20.3%)	10 (1.52%)	.756(1)	.385
disagree	94 (79.7%)	56 (84.8%)		
Statement 27: I do not easily express criticism of my colleagues or supervisor				
	pre	post	value (df)	p-value
agree	54 (45.8%)	25 (37.9%)	1.074(1)	.300
disagree	64 (54.2%)	41 (62.1%)		
Statement 28: When I do not agree with the way a colleague works, I say so				
	pre	post	value (df)	p-value
agree	96 (81.4%)	58 (87.9%)	1.320(1)	.251
disagree	22 (18.6%)	8 (12.1%)		
Statement 29: When I am the only one to disagree with the rest, I just keep quiet				
	pre	post	value (df)	p-value
agree	32 (27.1%)	23 (34.8%)	1.207(1)	.272
disagree	86 (72.9%)	43 (65.2%)		
Statement 30: When I do not agree with something at work, I find it hard to say so				
	pre	post	value (df)	p-value
agree	44 (37.3%)	31 (47%)	1.643(1)	.200
disagree	74 (62.7%)	35 (53%)		
Statement 31: I like to work with solid ideas and methods				
	pre	post	value (df)	p-value
agree	96 (82.1%)	58 (87.9%)	1.075(1)	.300
disagree	21 (17.9%)	8 (12.1%)		
Statement 32: I feel comfortable with working routines				
	pre	post	value (df)	p-value
agree	82 (70.1%)	50 (75.8%)	.675(1)	.411
disagree	35 (29.9%)	16 (24.2%)		
Statement 33: I do not like to deviate from prescribed methods				
	pre	post	value (df)	p-value
agree	46 (39.3%)	27 (40.9%)	.045(1)	.833
disagree	71 (60.7%)	39 (59.1%)		
Statement 34: I like to try things out, even if it sometimes leads nowhere				
	pre	post	value (df)	p-value*
agree	92 (78.6%)	56 (84.8%)	1.054(1)	.305
disagree	25 (21.4%)	10 (15.2%)		
Statement 35: I experiment with other working methods				
	pre	post	value (df)	p-value
agree	94 (80.3%)	55 (83.3%)	.250(1)	.617
disagree	23 (19.7%)	11 (16.7%)		
Statement 36: I try out new working methods				
	pre	post	value (df)	p-value
agree	99 (84.6%)	61 (92.4%)	2.342(1)	.126
disagree	18 (15.4%)	5 (7.6%)		
Statement 37: If I do not know what I really should know, I try to hide the fact				
	pre	post	value (df)	p-value
agree	15 (12.8%)	10 (15.2%)	.194(1)	.659

disagree	102 (87.2%)	56 (84.8%)		
Statement 38: I do not mind making mistakes				
	pre	*post*	*value (df)*	*p-value*
agree	70 (59.8%)	38 (57.6%)	.089(1)	.766
disagree	47 (40.2%)	28 (42.4%)		
Statement 39: If I have not done something very well, I prefer to keep quiet about it				
	pre	*post*	*value (df)*	*p-value**
agree	20 (17.1%)	10 (15.2%)	.116(1)	.733
disagree	97 (82.9%)	56 (84.8%)		
Statement 40: If people at work see that I am doing something wrong, I have the feeling that I have lost face				
	pre	*post*	*value (df)*	*p-value*
agree	32 (27.4%)	24 (36.4%)	1.614(1)	.204
disagree	85 (72.6%)	42 (63.6%)		
Statement 41: If I make a mistake, I find it hard to forgive myself				
	pre	*post*	*value (df)*	*p-value*
agree	50 (42.7%)	41 (62.1%)	6.344(1)	.012
disagree	67 (57,3%)	25 (37.9%)		
Statement 42: If I have not done something well, I try to forget about it as soon as possible				
	pre	*post*	*value (df)*	*p-value*
agree	38 (32.5%)	14 (21.2%)	2.633(1)	.105
disagree	79 (67.5%)	52 (78.8%)		
Statement 43: I get embarrassed if I make a mistake				
	pre	*post*	*value (df)*	*p-value*
agree	39 (33.3%)	22 (33.3%)	.000(1)	1.000
disagree	78 (66.7%)	44 (66.7%)		
Statement 44: I am consciously occupied with my career				
	pre	*post*	*value (df)*	*p-value**
agree	98 (83.8%)	56 (84.8%)	.037(1)	.847
disagree	19 (16.2%)	10 (15.2%)		
Statement 45: I think it is important to have a job in which I can develop				
	pre	*post*	*value (df)*	*p-value*
agree	105 (89.7%)	61 (92.4%)	.360(1)	.549
disagree	12 (10.3%)	5 (7.6%)		
Statement 46: I think about what sort of work I would like to be doing in five years' time.				
	pre	*post*	*value (df)*	*p-value*
agree	92 (78.6%)	58 (87.9%)	2.441(1)	.118
disagree	25 (21.4%)	8 (12.1%)		
Statement 47: I am continually occupied with my career development				
	pre	*post*	*value (df)*	*p-value*
agree	98 (83.8%)	60 (90.9%)	1.828(1)	.176
disagree	19 (16.2%)	6 (9.1%)		

** Fisher exact test when chi-square test was not possible*

Appendix 2: Survey Interprofessional Collaboration Measurement Scale – Nursing professionals

Statement 1: The nursing team has a good understanding with the allied/medical about the respective responsibilities

	pre	*post*	*value (df)*	*p-value*
agree	60 (83.3%)	31 (77.5%)	.574(1)	.449
disagree	12 (16.7%)	9 (22.5%)		

Statement 2: The allied/medical team is usually willing to take into account the convenience of the nursing team when planning their work

	pre	*post*	*value (df)*	*p-value*
agree	49 (69%)	30 (75%)	.447(1)	.504
disagree	22 (31%)	10 (25%)		

Statement 3: I feel that patient treatment and care are not adequately discussed between the nursing team and allied/medical team

	pre	*post*	*value (df)*	*p-value*
agree	15 (21.1%)	9 (22.5%)	.028(1)	.866
disagree	56 (78.9%)	31 (77.5%)		

Statement 4: The nursing team and allied/medical team share similar ideas about how to treat patients

	pre	*post*	*value (df)*	*p-value*
agree	45 (64.3%)	26 (65%)	.000(1)	.940
disagree	25 (35.7%)	14 (35%)		

Statement 5: The allied/medical team is willing to discuss nursing team issues

	pre	*post*	*value (df)*	*p-value**
agree	67 (95.7%)	38 (95%)		1.000
disagree	3 (4.3%)	2 (5%)		

Statement 6: The allied/medical team cooperates with the way we (nursing team) organise care

	pre	*post*	*value (df)*	*p-value*
agree	52 (74.3%)	32 (80%)	.460(1)	.497
disagree	18 (25.7%)	8 (20%)		

Statement 7: The allied/medical team would be willing to cooperate with new nursing team practices

	pre	*post*	*value (df)*	*p-value*
agree	61 (87.1%)	33 (82.5%)	.441(1)	.506
disagree	9 (12.9%)	7 (17.5%)		

Statement 8: The allied/medical team does not usually ask for our (nursing team) opinions

	pre	*post*	*value (df)*	*p-value*
agree	18 (25.7%)	5 (12.5%)	2.688(1)	.101
disagree	52 (74.3%)	35 (87.5%)		

Statement 9: The allied/medical team anticipates when the nursing team needs their help

	pre	*post*	*value (df)*	*p-value*
agree	40 (57.1%)	27 (67.5%)	1.147(1)	.284
disagree	30 (42.9%)	13 (32.5%)		

Statement 10: Important information is always passed on between the nursing team and the allied/medical team

	pre	*post*	*value (df)*	*p-value*
agree	57 (81.4%)	31 (77.5%)	.246(1)	.620
disagree	13 (18.6%)	9 (22.5%)		

Statement 11: Disagreements with the allied/medical team often remains unresolved				
	pre	*post*	*value (df)*	*p-value*
agree	16 (22.9%)	10 (25%)	.065(1)	.799
disagree	54 (77.1%)	30 (75%)		

Statement 12: The allied/medical team thinks their work is more important than the work of the nursing team				
	pre	*post*	*value (df)*	*p-value*
agree	6 (8.6%)	5 (12.5%)	.437(1)	.509
disagree	64 (91.4%)	35 (87.5%)		

Statement 13: The allied/medical team is not be willing to discuss their new practices with the nursing team				
	pre	*post*	*value (df)*	*p-value*[*]
agree	9 (12.9%)	3 (7.5%)		.530
disagree	61 (87.1%)	37 (92.5%)		

** Fisher exact test when chi-square test was not possible*

Appendix 3 – Survey Interprofessional Collaboration Measurement Scale – Allied/Medical professionals

Statement 1: The allied/medical team has a good understanding with the nursing team about the respective responsibilities				
	pre	*post*	*value (df)*	*p-value*
agree	27 (60%)	14 (66.7%)	.270(1)	.603
disagree	18 (40%)	7 (33.3%)		
Statement 2: The nursing team is usually willing to take into account the convenience of the allied/medical team when planning their work				
	pre	*post*	*value (df)*	*p-value**
agree	42 (93.3%)	18 (85.7%)		.373
disagree	3 (6.7%)	3 (14.3%)		
Statement 3: I feel that patient treatment and care are not adequately discussed between the allied/medical team and the nursing team				
	pre	*post*	*value (df)*	*p-value*
agree	16 (35.6%)	1 (4.8%)	7.100(1)	.000
disagree	29 (64.4%)	20 (95.2%)		
Statement 4:The allied/medical team and nursing team share similar ideas about how to treat patients				
	pre	*post*	*value (df)*	*p-value*
agree	28 (62.2%)	14 (66.7%)	.122(1)	.727
disagree	17 (37.8%)	7 (33.3%)		
Statement 5: The nursing team is willing to discuss allied/medical team issues				
	pre	*post*	*value (df)*	*p-value**
agree	35 (77.8%)	18 (85.7%)		.526
disagree	10 (22.2%)	3 (14.3%)		
Statement 6: The nursing team cooperates with the way we (allied/medical team) organise care				
	pre	*post*	*value (df)*	*p-value**
agree	35 (77.8%)	18 (85.7%)		.526
disagree	10 (22.2%)	3 (14.3%)		
Statement 7: The nursing team would be willing to cooperate with new allied/medical team practices				
	pre	*post*	*value (df)*	*p-value**
agree	39 (86.7%)	17 (81%)		.714
disagree	6 (13.3%)	4 (19%)		
Statement 8: The nursing team does not usually ask for our (allied/medical team) opinions				
	pre	*post*	*value (df)*	*p-value*
agree	10 (22.2%)	7 (33.3%)	.924(1)	.336
disagree	35 (77.8%)	14 (66.7%)		
Statement 9: The nursing team anticipates when the allied/medical team needs their help				
	pre	*post*	*value (df)*	*p-value*
agree	29 (64.4%)	12 (57.1%)	.324(1)	.569
disagree	16 (35.6%)	9 (42.9%)		
Statement 10: Important information is always passed on between the allied/medical team and the nursing team				
	pre	*post*	*value (df)*	*p-value*
agree	24 (53.3%)	17 (81%)	4.642(1)	.031
disagree	21 (46.7%)	4 (19%)		

Statement 11: Disagreements with the nursing team often remains unresolved				
	pre	*post*	*value (df)*	*p-value*
agree	16 (35.6%)	5 (23.8%)	.911(1)	.340
disagree	29 (64.4%)	16 (76.2%)		
Statement 12: The nursing team thinks their work is more important than the work of the allied/medical team				
	pre	*post*	*value (df)*	*p-value*
agree	5 (11.1%)	1 (4.8%)		.656*
disagree	40 (88.9%)	20 (95.2%)		
Statement 13: The nursing team is not be willing to discuss their new practices with the allied/medical team				
	pre	*post*	*value (df)*	*p-value**
agree	2 (4.4%)	2 (9.5%)		.587
disagree	43 (95.6%)	19 (90.5%)		

** Fisher exact test when chi-square test was not possible*
