# Supplementary material for: Which actions contribute to the development of an interprofessional learning and working culture in nursing homes? A realist action study
Source: BMJ Open. 2024 Sep 20;14(9):e085096. doi: 10.1136/bmjopen-2024-085096 (PMC11418486; doi:10.1136/bmjopen-2024-085096)
Supplement: Supplementary file 2 [file bmjopen-2024-085096.reviewer_comments.pdf]

PEER REVIEW HISTORY

BMJ Open publishes all reviews undertaken for accepted manuscripts. Reviewers are asked to complete a checklist review form and are provided with free text boxes to elaborate on their assessment. These free text comments are reproduced below.

ARTICLE DETAILS

**TITLE (PROVISIONAL)**	Which actions contribute to the development of an interprofessional learning and working culture in nursing homes? A realist action study.
**AUTHORS**	Verbeek, Frank; Nouwens, Elvira S.; Lovink, Marleen; Huisman-de Waal, Getty; Fluit, Cornelia; Laurant, Miranda; van Vught, Anneke

VERSION 1 - REVIEW

**REVIEWER NAME**	***Rutten, Johanna***
**REVIEWER AFFILIATION**	Rutten, Johanna
**REVIEWER CONFLICT OF INTEREST**	Na
**DATE REVIEW RETURNED**	19-Feb-2024

**GENERAL COMMENTS**	Thank you for letting me review this manuscript. It provides interesting new knowledge on interprofessional learning. I would be curious how the identified actions affect the person-centered care experienced by the residents, but of course this would exceed the purpose of this manuscript.

**REVIEWER NAME**	***Nakrem, Sigrid***
**REVIEWER AFFILIATION**	Norwegian University of Science and Technology, Department of Public Health and Nursing
**REVIEWER CONFLICT OF INTEREST**	Na
**DATE REVIEW RETURNED**	04-Mar-2024

**GENERAL COMMENTS**	Thank you for the opportunity to review this manuscript. The paper is reporting a rather complex and "messy" project, and it is not easy to get a good overview of the rationale for the study, and the reason for choosing the methodological approach (realist action research). It seems that the mix of both action research and realist evaluation is the reason why it is challenging to review the paper. Only after reading the published protocol, I did understand the full scope of the project. In my opinion, readers should be able to read the paper as a stand-alone paper.<br><br>I suggest that the introduction should be more to the point about what the problem is (other than the increased population of older people needing complex care), and that the connection between person-centered care (or quality of care/patient safety?) and an interprofessional working culture is elaborated. Person-centered care needs to be defined, as this can mean different things, especially in nursing homes where the patients/residents are individuals with a variety of diagnosis and complex needs. The

	methods used in the research, and in particular analytical approach is not easy to grasp, and I suggest that figure 1 is enhanced to give more details, including a timeline and participating actors. Both figure 2 and 3 suggest that there are linear connections between actions and context, while it is not clear how actions actually influenced the context (I suppose the context is relatively stable structures, while actions are those introduced during the project?).<br><br>It is not clear whether the study related to developing an Evidence-Based Nursing Culture in nursing homes is part of (or the same?) study as the present study about interprofessional working culture. Please clarify.<br><br>Further the following should be considered:<br>- What is meant by "management with a broad vision on interprofessional working? (In the abstract and on page 13, line 29). Please define or clarify.<br>- Include more up to date literature on interprofessional learning and working culture (some of the literature that is referred to are more than 15 years old)<br>- Not all readers would be familiar with the European qualification framework (EQF) and the levels. I suggest that you explain what the different levels mean in the context of Dutch nurses/allied health care professionals (i.e. vocational nurses, RNs, bachelor or master prepared healthcare professionals etc.). Please add a note to table 1 explaining the levels.<br>- In Step 2, individual interviews were performed, how were these interviews analysed and used in this paper when presenting the results?<br>- What Covid-19 measures were evident? The sentences on page 9, lines 47-53 are not clear. Please elaborate.<br>- In table 2 the authors mention "posters", what is this? Please explain.<br>- Please do not use the abbreviation CMO without spelling it out the first time (page 10).<br>- In the discussion the authors state "We have learned..." several times, however, it is not clear whether it is the researchers that learned something, or the nursing home staff/leaders. Please clarify.<br>- What is an electronic patient dossier? Do you mean medical record or other application with all patient information?<br>- Did the internal coach participate in the research group or in any of the interviews? And who was the action researcher (page 7, line 47). If the authors improve figur 1 to also include the different actors in the research, it would be easier to explain who participated in the study.<br>- There are a few spelling and grammar errors, please proof read the paper thoroughly.

**REVIEWER NAME**	***Chadborn, Neil***
**REVIEWER AFFILIATION**	University of Nottingham, School of Medicine
**REVIEWER CONFLICT OF INTEREST**	na
**DATE REVIEW RETURNED**	17-Mar-2024

**GENERAL COMMENTS**	This manuscript describes an important aspect of care for residents of nursing homes, but the authors do not clearly describe the need or research gap. Whilst the research describes in detail the various contextual factors and mechanisms, only one general outcome is

	presented, which seems to lack nuance or specificity. The intervention or change project does not seem to be clearly described, beyond ‘coaching’ and ‘practice development’.<br>1. No specific details are given of care teams – it would help the reader to understand the context if the various teams were categorised – are some teams specific to one care home, do other teams visit several care homes<br>2. No detail of coaching – not always clear when text mentions ‘coach’ whether this refers to external or internal coach<br>Therefore it is unclear what is the specific nature of the intervention or change process that is being evaluated.<br>Text refers to patterns of CMOs but these are not described in CMO format – just a list of mechanisms and the only outcome is ‘interprofessional learning etc’. However Appendix 1 does list many outcomes – It would be useful to see these summarised in the text.<br>“Step 1 – Plan” states that the study started by formulating initial theory (p6 line 20) – who was involved in this formulation – the author team or also the internal coaches or teams?<br>The manuscript mentions “successful topics” p7, line 43 – who defined success? Were the teams consulted on what constituted “success”.<br>P11 line 47 discusses evidence-based projects – again it is unclear who initiates or is involved in these – eg the whole team? However the quote indicates the importance of being involved in research. So this also seems like an important outcome, as well as the stated outcome of interdisciplinary learning.<br>The abstract doesn’t describe the nature of the problem or give a background to the study.

VERSION 1 – AUTHOR RESPONSE

Reviewer’s comments

#1 reviewer – Dr. Johanna Rutten

Thank you for letting me review this manuscript. It provides interesting new knowledge on interprofessional learning. I would be curious how the identified actions affect the person-centered care experienced by the residents, but of course this would exceed the purpose of this manuscript.

Response:

Thank you for your feedback on our manuscript. Your question about how the identified actions affect the person-centred care experiences by residents was as such not included in our study. However, this would be a great suggestion for further research. Therefore, we have added the following text to the Discussion (page 17):

‘We were interested in the actions to develop an interprofessional learning and working culture. We saw that these actions also improved person-centred care in some way. However, we were not able to measure the effect of person-centred care in this study. Therefore, we

suggest further research to find how the actions affect the person-centred care experienced by the residents in the nursing homes.'

#2 reviewer – Dr. Sigrid Nakrem

Thank you for the opportunity to review this manuscript. The paper is reporting a rather complex and "messy" project, and it is not easy to get a good overview of the rationale for the study, and the reason for choosing the methodological approach (realist action research). It seems that the mix of both action research and realist evaluation is the reason why it is challenging to review the paper. Only after reading the published protocol, I did understand the full scope of the project. In my opinion, readers should be able to read the paper as a stand-alone paper

***Response*:**

Thank you for your constructive and important feedback. We agreed that readers should be able to read the paper on its own. Therefore, we have revised our manuscript based on your suggestions. We have revised the introduction, the argumentation of the design and the first figure in the paper.

I suggest that the introduction should be more to the point about what the problem is (other than the increased population of older people needing complex care), and that the connection between person-centred care (or quality of care/patient safety?) and an interprofessional working culture is elaborated.

Response:

We have revised the first two paragraphs of the introduction (page 4):

'In the Netherlands, most older people receive care at home from primary healthcare professionals and informal caregivers. When it is no longer possible to receive this care at home, people with more complex needs are admitted to nursing homes. To meet more complex needs, it is important to work together in an interprofessional way to provide the best quality of person-centred care for nursing home residents. In person-centred care, the residents and their families become partners with professionals in their own care. The focus is on shared decision-making, emotional well-being and personal goals of the resident, rather than on the illness.

Delivering person-centred care requires intensive collaboration between the resident, their relatives, their informal network and the usually many professionals involved. In the Netherlands, professionals in a nursing home are organised into separated teams, the nursing professionals and allied/medical healthcare professionals. Nursing teams are a mix of nurse

aides, nurse assistants, licensed practical nurses, vocationally trained registered nurses and baccalaureate-educated registered nurses at levels 1-6 of the European Qualification Framework. Allied/medical healthcare professionals include physiotherapists, dieticians, speech therapists and general or elderly care physicians. Each professional has their own expertise and competencies in the care for residents.'

Person-centred care needs to be defined, as this can mean different things, especially in nursing homes where the patients/residents are individuals with a variety of diagnosis and complex needs.

Response:

We have added the following explanation in the introduction (page 4):

'In person-centred care, the residents and their families become partners with professionals in their own care. The focus is on shared decision-making, emotional well-being and personal goals of the resident, rather than on the illness.'

The methods used in the research, and in particular analytical approach is not easy to grasp, and I suggest that figure 1 is enhanced to give more details, including a timeline and participating actors. Both figure 2 and 3 suggest that there are linear connections between actions and context, while it is not clear how actions actually influenced the context (I suppose the context is relatively stable structures, while actions are those introduced during the project?).

Response:

We understand that the methods of an analytical approach are not easy to grasp. To provide a better overview, we decided to change figure 1 and to add one more step instead of three. Hopefully, this gives a better overview of the different steps and methods we used. We decided not to change figures 2 and 3 because the context isn't stable. Therefore, each participating team used or created different actions. This allowed them to tailor the actions to their context.

'We now mention four steps in our manuscript: 1) formulating theory, 2) insights about cultural elements, 3) act & observe and 4) reflect' (page 4).

It is not clear whether the study related to developing an Evidence-Based Nursing Culture in nursing homes is part of (or the same?) study as the present study about interprofessional working culture. Please clarify.

Response:

The study about the development of an Evidence-Based Nursing Culture in nursing homes is not a part of this study. But we used the results of the EBN nursing home study in our initial theory. To clarify this, we have added the following sentence (page 5).

'This initial theory was based on a comprehensive literature search and a previous action research in nursing homes about the development of an evidence-based nursing culture in nursing teams.'

Further the following should be considered:

- **What is meant by "management with a broad vision on interprofessional working? (In the abstract and on page 13, line 29). Please define or clarify.**

Response:
We have changed this sentence to 'management supporting interprofessional working' and added an explanation: 'When management provided support for interprofessional working, such as setting goals for interprofessional working, making time for interprofessional working, discussing interprofessional working and improving interprofessional working, the team felt there was a real opportunity to work together.' (page 14)

- **Include more up to date literature on interprofessional learning and working culture (some of the literature that is referred to are more than 15 years old)**

Response:
We have updated the reference-list, see red-marked references on pages 18, 19 and 20.

- **Not all readers would be familiar with the European qualification framework (EQF) and the levels. I suggest that you explain what the different levels mean in the context of Dutch nurses/allied health care professionals (i.e. vocational nurses, RNs, bachelor or master prepared healthcare professionals etc.). Please add a note to table 1 explaining the levels.**

Response:

We have added a note to Table 1 with an explanation of the levels (page 9).

- **In Step 2, individual interviews were performed, how were these interviews analysed and used in this paper when presenting the results?**

Response:

The results of the interviews were only summarised and highlighted for the kick-off meetings (step 2) and were not used in the data-analyses. For a better overview of this process we have added a new Figure 1 and a new step with more explanation 'step 2 – insights about cultural elements.' (page 6)

- **What Covid-19 measures were evident? The sentences on page 9, lines 47-53 are not clear. Please elaborate.**

Response:
We have added the following sentence: 'In the Netherlands, all nursing homes for the elderly were closed to visitors, family and certain allied/medical healthcare professionals who were not involved in the basic care for the elderly. This measure played a major role in interprofessional learning and working, because there were fewer physical meetings with the interprofessional teams, and less attention given to interprofessional working and more to the COVID-19 pandemic.' (page 9)

- **In table 2 the authors mention "posters", what is this? Please explain.**

Response:
We have changed the sentence to: '*Using post-it notes to understand what people think the tasks and responsibilities of each profession are.' (Table 2)*

And to: *'Making ideas transparent about what subjects motivate people and what we can develop through using post-it notes on flip charts' (Table 3)*

- **Please do not use the abbreviation CMO without spelling it out the first time (page 10)**

Response:

We decided to use the full (context-mechanism-outcome) term in this paper.

- **In the discussion the authors state "We have learned..." several times, however, it is not clear whether it is the researchers that learned something, or the nursing home staff/leaders. Please clarify.**

Response:

With this sentence, we want to explain that the action research, the coach and professionals saw this during the study. To prevent confusion, we have deleted the sentence 'we have learned' in the Discussion and Abstract.

- **What is an electronic patient dossier? Do you mean medical record or other application with all patient information?**

Response:

We meant an electronic patient record. We have changed electronic patient dossier to electronic patient record.

- **Did the internal coach participate in the research group or in any of the interviews? And who was the action researcher (page 7, line 47). If the authors improve figur 1 to also include the different actors in the research, it would be easier to explain who participated in the study.**

Response:

The internal coaches did not participate in the research group, but during the research we had some meetings with all internal and external coaches to share ideas, actions and to discuss the process.

We have added the following sentence: 'During the action and observation period we had several meetings with the internal coaches, the external coaches and the researcher (FV) to share ideas, inspiration and discuss the process.' (page 7)

The researcher was the first author of this study. To give better insight about which person was involved we have added abbreviations of the name of the persons.

- **There are a few spelling and grammar errors, please proof read the paper thoroughly.**

Response:

We have proofread the paper.

#3 reviewer – Dr. Neil Chadborn

This manuscript describes an important aspect of care for residents of nursing homes, but the authors do not clearly describe the need or research gap. Whilst the research describes in detail the various contextual factors and mechanisms, only one general outcome is presented, which seems to lack nuance or specificity. The intervention or change project does not seem to be clearly described, beyond ‘coaching’ and ‘practice development’.

Response:

Thank you for your constructive feedback. We agreed that the need or research gap was not clearly described. We have added the following text to the last paragraph of the introduction (page 4):

‘Interprofessional learning and working leads to high standards of care for residents, exemplified by an increase in optimal processes, motivated professionals and involvement of residents and their families in care. However, there is a gap of knowledge about what works, in what context and in what manner it works in daily nursing home practices. There is therefore a need to identify which actions contribute to the development of an interprofessional learning and working culture in nursing homes and to what extent.’

Further, our aim was to identify the specific context and actions that trigger mechanisms for the development of an interprofessional learning and working culture. Therefore, we described in more detail the various contextual factors and mechanisms instead of the outcome. To provide more detail about our outcome (the development of an interprofessional learning and working culture), we have added the following text in the summary of the initial theory:

‘The interprofessional learning and working culture is a culture in which at least two healthcare professionals collaborate and learn together. Innovating, communicating, keeping each other informed, being aware of each other, sharing compliments, sharing successes and collaborating with the residents and their families are needed in this culture. Outcomes could affect at resident, team and organisational level.’ (page 6)

The intervention is not described in detail, because we have also published a research protocol. In this protocol we described in more detail the nine principles of practice development and the cyclic process. Coaching is one of these principles. To provide more detail, we have added the following text in the paragraph under the heading ‘Act and Observe’:

‘The cyclic process consisted of six processes (called PDDOEN in Dutch): 1) creating an overview on a subject, the need, the why and who are involved, 2) setting a goal to improve interprofessional learning and working, 3) selecting or creating the needed actions, 4) sharing and collecting new insights, 5) evaluating and 6) selecting/creating actions or selecting a new topic.’ (page 7)

1. **No specific details are given of care teams – it would help the reader to understand the context if the various teams were categorised – are some teams specific to one care home, do other teams visit several care homes**

Response:

Each team was working on one unit in the nursing home. None of the teams visited several care homes. In the Netherlands, teams work in one specific department. Only allied/medical healthcare professionals can work in more than one unit. To give more information about the team in the Netherlands, we have added the following sentence under the heading 'participants':

'In the Netherlands, each unit in a nursing home, such as a geriatric rehabilitation unit, has a nursing team. Usually only allied/medical healthcare professionals work on more than one unit. The allied/medical healthcare professionals were included in the study when they provide care to residents on the participating unit.' (page 5)

2. **No detail of coaching – not always clear when text mentions 'coach' whether this refers to external or internal coach Therefore it is unclear what is the specific nature of the intervention or change process that is being evaluated.**

Response:

Thank you for this feedback. To specify, we have added 'internal' and/or 'external' coaching to the text.

As such, we have added more information about the internal and external coach in the text:

Internal coach:

'The internal coach or coaches per participating team were selected for their motivation, leadership skills and reflective capacity to coach an interprofessional team in working and learning together in an interprofessional way.' (page 7)

External coach:

'The external coaches were experienced lecturers in nursing and allied/medical healthcare and had expertise in culture change, the nursing home setting and interprofessional learning and working.' (page 7)

3. **Text refers to patterns of CMOs but these are not described in CMO format – just a list of mechanisms and the only outcome is 'interprofessional learning etc'. However Appendix 1 does list many outcomes – It would be useful to see these summarised in the text.**

Response: the relationship between the actions and outcomes is presented under the heading 'Outcome' in Results in the two themes. To give a better overview, we have added the outcomes of the tables from the appendix to table 2 and 3 in the paper. We decided to present our results not in specific patterns, because our interests were the actions for the development of an interprofessional learning and working culture. Therefore, we chose to present the results in a list of actions, context factors, mechanisms and outcomes.

4. **"Step 1 – Plan" states that the study started by formulating initial theory (p6 line 20) – who was involved in this formulation – the author team or also the internal coaches or teams?**

   Response:
   We have added the following sentence under the heading 'Formulating theory':

'This theory was formulated with experts on interprofessional learning and working, the project leader, the researcher and two external coaches.' (page 5)

5. **The manuscript mentions "successful topics" p7, line 43 – who defined success? Were the teams consulted on what constituted "success".**

Response:

We decided to delete 'successful' in this sentence to specify that the teams identified topics to work on with the internal coach. We have now changed this sentence to:

'Highlights of the questionnaires, interviews and the discussion in the kick-off meeting were subsequently used to identify topics for the development of an interprofessional learning and working culture.' (page 7)

6. **P11 line 47 discusses evidence-based projects – again it is unclear who initiates or is involved in these – eg the whole team? However the quote indicates the importance of being involved in research. So this also seems like an important outcome, as well as the stated outcome of interdisciplinary learning.**

Response:

We agreed that it is unclear who is involved in these. We have added the following sentence to the text, page XX

‘The facilitation in space and time to be a champion or to learn and work together as an interprofessional team on projects, for example…’ (page 11)

Being involved in research is also an important outcome. Under the heading ‘outcome’ we mentioned this: ‘Professionals were innovating, doing research, sharing information and sharing compliments and successes.’ (page 12)

7. **The abstract doesn’t describe the nature of the problem or give a background to the study.**

Response:

We did not describe the nature of the problem of the study because of the structure and guidelines from BMJ Open. We only mentioned the study objective. But to provide more insight, we have added the following sentence to the objective in the abstract:

‘Insights about what actions contributed to the development of an interprofessional learning and working culture were lacking for nursing homes.’ (page 2)

VERSION 2 – REVIEW

**REVIEWER NAME**	***Rutten, Johanna***
**REVIEWER AFFILIATION**	Maastricht University
**REVIEWER CONFLICT OF INTEREST**	Na
**DATE REVIEW RETURNED**	25-Jun-2024

**GENERAL COMMENTS**	Thank you for revising the manuscript and considering the reviewers' comments. The paper is improved. I would recommend to look at parts of the discussion section again. In particular try to avoid statements such as 'we learned that...' and 'the study has given us a better understanding...'; or 'we saw that....', since it might be unclear who you are referring to (the authors/researchers and/or the participants etc).

**REVIEWER NAME**	***Chadborn, Neil***
**REVIEWER AFFILIATION**	University of Nottingham, School of Medicine

**REVIEWER CONFLICT OF INTEREST**	Na
**DATE REVIEW RETURNED**	11-Jul-2024

**GENERAL COMMENTS**	Thanks to the authors for responding clearly to reviewer's comments and for amendments which have improved clarity of the manuscript. I have remaining concerns or queries which I hope can further improve several aspects of the paper:<br><br>Helpful clarification that the purpose was to improve person-centred care. “In person-centred care, the residents and their families become partners with professionals in their own care. The focus is on shared decision-making,”<br>I am still unclear as to the nature of the teams involved in the action research. Bearing in mind the above statement – were residents or family members involved in project meetings and were they participants in action research? Whilst residents and family are mentioned in the manuscript and protocol, it is difficult to see their views reflected in the data, or even see their details in the tables of participants.<br>Results – demographic data section starts with the confusing statement: “In total, 128 (66%) professionals completed the two questionnaires at the start of the research and 66 (34%) at the end of the research.” If 128 participants are only 66% of the participants – who are the other 34%. I can’t see these described in the table. I can’t see any reference to residents and family members.<br>So many data sources – but where are these data reported – Interviews? Observations? Is it reported elsewhere? What is the connection between these different sources of data and the themes, actions and mechanisms presented here?<br>To what extent did (internal) coaches influence the interprofessional learning. I would like to know whether internal coaches were generally nurses or allied health professionals / doctors. I’m surprised that the role of the internal coach does not seem to be mentioned within the realist analysis. Maybe I should assume that internal coaches led the daily evaluations (Table 3)? However my critical interpretation of this absence of data is that the internal coaches role was minimal in terms of coaching or leadership. If so, this may be an important finding.<br><br>Minor amendments:<br>P17 A lot of students are participating as interns in such units for several months and work on various professional and interprofessional assignments

VERSION 2 – AUTHOR RESPONSE

#2 reviewer – Dr. Sigrid Nakrem

Thank you for revising the manuscript and considering the reviewers' comments. The paper is improved. I would recommend to look at parts of the discussion section again. In particular try to avoid statements such as 'we learned that...' and 'the study has given us a better understanding...'; or 'we saw that....', since it might be unclear who you are referring to (the authors/researchers and/or the participants etc).

Response:

We agree that statements such as ‘we learned…’, ‘the study has given…’ or ‘we saw that…’ are unclear. Therefore, we decided to revise these sentences in the discussion and the conclusion part. See red marked changes on pages 16-19.

#3 reviewer – Dr. Neil Chadborn

Thank you for your constructive feedback and remaining queries to improve several aspects of the manuscript.

Helpful clarification that the purpose was to improve person-centred care. “In person-centred care, the residents and their families become partners with professionals in their own care. The focus is on shared decision-making,” I am still unclear as to the nature of the teams involved in the action research. Bearing in mind the above statement – were residents or family members involved in project meetings and were they participants in action research? Whilst residents and family are mentioned in the manuscript and protocol, it is difficult to see their views reflected in the data, or even see their details in the tables of participants.

Response:

Indeed, we are unclear on how we involved residents or family members in the project. The project focused on an interprofessional learning and working culture encouraging professionals to carry out actions to deliver more person-centered care. In line with the practice development (PD) approach, residents and families were partners in the project. More specific in selecting and performing actions to encourage interprofessional working contributing to person-centered care. To mention the focus on improving person-centered care, we decided to describe this in the method section where we give more information about the nine principles of the practice development approach, see page 8.

‘These coaches worked with the practice development approach (PD) to support and encourage professionals to work together and to involve residents and family members. Residents and family members, together with professionals, selected and performed the actions to encourage interprofessional working contributing to person-centered care. The PD approach focused on creating working and learning cultures and on developing person-centred cultures. The PD approach consisted of nine principles…’

Results – demographic data section starts with the confusing statement: “In total, 128 (66%) professionals completed the two questionnaires at the start of the research and 66 (34%) at the end of the research.” If 128 participants are only 66% of the participants – who are the other 34%. I can’t see these described in the table.

Response:

Thank you for this feedback. This section is confusing, and we decided to delete the percentages in this statement, see page 9.

I can't see any reference to residents and family members.

Response:

See the response about the involvement of residents and family members.

So many data sources – but where are these data reported – Interviews? Observations? Is it reported elsewhere? What is the connection between these different sources of data and the themes, actions and mechanisms presented here?

Response:

In line with the design, we used different sources of data to answer the research question. The data of the interviews was only used for the kick-off meetings as now described more in the methods section (page 7).

'Highlights of these summaries were recorded to provide an overall picture of the interprofessional learning and working culture in each participating team. These highlights were only used to present and discuss in the kick-off meetings in each participating team (see step 3).'

The questionnaires were used for the kick-off meetings and to evaluate the differences in reflective work behaviour (mechanism) and interprofessional working (outcome). The logbooks were used to get an overview about the performed actions. The data of the focus group interviews was used to create insight into the actions, the influence of the context and how these actions worked. We transcribed the focus groups and analysed the transcriptions in Atlas Ti. The connection between these different sources is described in 'Step 4 – Reflect'. 'Firstly, we identified the actions by screening the logbooks. Secondly, we discussed the actions and the influence of the context and how these actions worked in the focus group interviews. Lastly, we discussed patterns of what works, in what context and how with a group of experts and formulated these results in themes of actions, including the way they work (context, mechanism and outcome) in the paper.'

To what extent did (internal) coaches influence the interprofessional learning. I would like to know whether internal coaches were generally nurses or allied health professionals / doctors. I'm surprised that the role of the internal coach does not seem to be mentioned within the

realist analysis. Maybe I should assume that internal coaches led the daily evaluations (Table 3)? However, my critical interpretation of this absence of data is that the internal coaches role was minimal in terms of coaching or leadership. If so, this may be an important finding.

Response:

Good point, thank you. The coaches were key in selecting actions and encouraging professionals to work in an interprofessional way (see page 8).

These coaches worked with the practice development approach (PD) to support and encourage professionals to work together and to involve residents and family members. Residents and family members, together with professionals, selected and performed the actions to encourage interprofessional working contributing to person-centered care.

Internal coaches were nurses and allied healthcare professionals. This differs between the participating teams. To create more insight about the background of the coach we decided to mention this in the methods section (see page 8) and in the demographic data (see page 10)

Methods:

"The internal coaches were employees of the nursing team, the allied/medical healthcare team, or a combination of both teams."

Demographic data:

Fourteen internal coaches and two external coaches participated in this study. Two internal coaches in organisation 1 (belonging to the allied/medical staff), one internal coach in organisation 2 (belonging to the nursing staff), four internal coaches in organisation 3 (two belonging to the nursing staff and two belonging to the allied/medical staff), two in organisation 4 (one belonging to the nursing staff and one to the allied/medical staff), three in organisation 5 (two to the nursing staff and one to the allied/medical staff) and two internal coaches were participating in organisation 6 (belonging to the nursing staff).

The internal coaches were involved in the data analysis, as we analysed their logbooks, they participated in the focus group interviews, and they were expert in the group of experts (step 4 reflect). Further, the role of the internal coach was mentioned in theme 1 in the results section, but we used the term 'champignon there. We decided to revise this to 'the internal coach' in the text, see page 12.

At least, the focus of the study was on the actions that contribute to an interprofessional learning and working culture. Therefore, this study understudied the influence of personal characteristics, and the way works to promote interprofessional working and learning. We recommend further research to investigate the influence of the coaches on the

interprofessional learning and working culture in more detail. This recommendation is presented in the discussion part, see page 18.

'Furthermore, the influence of coaches' (personal) characteristics on the development of interprofessional learning culture was not investigated in this study. It is recommended for further research to investigate the (personal) characteristics of the coaches on interprofessional learning and working so that they can be considered when selecting and training coaches to develop an interprofessional learning and working culture.'

Minor amendments:
P17 A lot of students are participating as interns in such units for several months and work on various professional and interprofessional assignments.

Response:

Thank you. We changed this sentence.
